# Effect of resistance training with and without vitamin D calcium chitosan nanoparticle supplements on apoptosis markers in ovariectomized rats: An experimental study

**DOI:** 10.18502/ijrm.v20i7.11557

**Published:** 2022-08-08

**Authors:** Mahsa Dehghan-Manshadi, Mohammad-Ali Azarbayjani, Sirvan Atashak, Maghsoud Peeri, Saleh Rahmati-Ahmadabad

**Affiliations:** ^1^Department of Exercise Physiology, Central Tehran Branch, Islamic Azad University, Tehran, Iran.; ^2^Department of Exercise Physiology, Mahabad Branch, Islamic Azad University, Mahabad, Iran.; ^3^Department of Physical Education, Pardis Branch, Islamic Azad University, Pardis, Iran.

**Keywords:** Apoptosis, BCL-2-associated X protein, Caspase-3, Estrogen replacement therapy, Hormone replacement therapy.

## Abstract

**Background:**

Hormone therapy is one of the most effective treatments for menopausal disorders, but it may increase the risk of breast cancer, coronary heart disease, and pulmonary embolism.

**Objective:**

The present study investigated the effect of resistance training with and without vitamin D calcium(Ca ${}^{++}$ ) chitosan nanoparticles on apoptosis markers in ovariectomized rats.

**Materials and Methods:**

42 female Wistar rats were divided into 7 groups (n = 6/each). One group was assigned as the healthy controls to show the induction of menopause. The other 6 groups comprised ovariectomized (OVX) animals including: 1) vitamin D + calcium + chitosan + resistance training, 2) saline + estrogen + resistance training, 3) saline + resistance training, 4) vitamin D + calcium + chitosan, 5) saline + estrogen, and 6) OVX + control. 48 hr after the last intervention, the hippocampus tissue was extracted to measure the BCL-2-associated X (*BAX*), B-cell lymphoma 2 (*BCL-2*), and caspase-3 gene expression as well as the percentage of dead cells.

**Results:**

OVX rats demonstrated increased *BAX* gene expression, ratio of *BAX* gene expression to *BCL-2*, caspase-3 gene expression, and percentage of dead cells of hippocampal tissue, but decreased *BCL-2* gene expression. Resistance training and vitamin D Ca ${}^{++}$ chitosan nanoparticle supplements seemed to reverse these changes.

**Conclusion:**

The combination of resistance training and vitamin D Ca ${}^{++}$ chitosan nanoparticle supplements may be considered a non-pharmacological treatment for OVX-induced apoptosis.

## 1. Introduction

Menopause is a period that occupies almost one-third of a woman's life, and hormonal changes cause a permanent interruption of menstruation (1). Estrogen deficiency in postmenopausal women leads to the release of free radicals and reactive oxygen species, causing oxidative stress, increases the risk of metabolic and other diseases, decreases bone density, and induces premature cell aging (2). It is abundantly clear that menopause can lead to mitochondrial genome mutations and cell death as a result of increased oxidative stress (2, 3). Essentially, apoptosis is the last phase of cellular damage, and researchers believe that menopause and aging are factors that contribute to apoptosis (4, 5). Caspase-3 is one of the most important factors associated with apoptosis, and its activation indicates irreversible cell apoptosis (6, 7). BCL-2-associated X (*BAX*) is a proapoptotic member of the B*-*cell lymphoma 2 (*BCL-2*) family, which normally resides in the cytosol (8). Apoptosis is determined by the ratio of *BAX* to *BCL-2*, an apoptotic and antiapoptotic protein, respectively (8).

Hormone therapy is one of the most effective treatments for menopausal disorders, but it may increase the risk of breast cancer, coronary heart disease, and pulmonary embolism (9, 10). Due to the side effects of hormone therapy in menopause, physical activity and use of supplements have received a lot of attention. Physical activity, especially resistance training, as a non-pharmacological method used to prevent and reverse the physiological changes caused by menopause, is recommended. Resistance training has been shown to have beneficial effects on the organs and systems of the body in postmenopausal women (11). Regular exercise training with appropriate intensity has been shown to cause structural and molecular changes (associated with inflammation factors such as *BAX*, *BCL-2*, caspase-3, and cell death) in muscle tissue and other tissues such as in the hippocampus (12, 13). Studies show that the use of supplements reduces menopausal disorders in women (14, 15).

Taking supplements such as vitamin D and calcium positively affects apoptosis in menopausal conditions. Vitamin D can modulate apoptosis-related components in menopausal conditions and can control cell death by inhibiting apoptotic proteins or stimulating anti-apoptotic proteins (16). Chitosan is a popular dietary fiber derived from the cuticles of crustaceans such as shrimp, crab, and lobster, or the cell wall of mushrooms (17). It has been shown that chitosan alleviates menopausal symptoms and modulates the gut microbiota in estrogen-deficient rats (18). The *BCL-2/BAX* pathway inhibits glutamate-induced cell death in *PC12* cells when chitosan oligosaccharides are acetylated (19). Insufficient information is available on the effect of vitamin D or chitosan intake on *BAX*, *BCL-2*, and caspase-3 in menopause conditions.

Experimentally, menopause can be mimicked by removing the ovaries, a procedure known as ovariectomy. To better understand the physiological phenomena and mechanisms associated with estrogen decline, ovariectomies are used as an experimental model.

Considering the above points, the present study aimed to investigate the effect of resistance training with and without vitamin D calcium chitosan nanoparticles on some apoptosis markers (*BAX*, *BCL-2*, caspase-3) in ovariectomized (OVX) rats.

## 2. Materials and Methods

### Animals

In this experimental study, 42 female Wistar rats were randomly selected and equally divided into 7 groups. One group was assigned as the healthy controls to show the induction of menopause. The other 6 groups comprised OVX animals including: 1) vitamin D + calcium + chitosan + resistance training, 2) saline + estrogen + resistance training, 3) saline + resistance training, 4) vitamin D + calcium + chitosan, 5) saline + estrogen, and 6) OVX + control.

### Induction of menopausal model

To create the model, 36 8-wk-old female rats were anesthetized using ketamine (30-50 mg/kg, Sigma-Aldrich, United States) and xylazine (3-5 mg/kg, Sigma-Aldrich, United States). The fur was then shaved from the lumbar region and the ovaries were surgically removed from the back of the rats. The wounds were then sutured, and the rats were kept for 1 month to induce the model. The rats were then grouped into the treatment groups of resistance training, supplementation, or simultaneous resistance training and supplementation.

### Training protocol

Rats were first introduced to the resistance training protocol (3 sessions per wk) on a ladder. Then they performed the resistance training protocol for 6 wk (intensity of exercise: 75-90% rat body weight, 6 repeats and 1 min rest between repetitions).

### Vitamin D calcium (Ca ${}^{++}$ ) chitosan supplementation

Chitosan** solution (1% w/v) was prepared by dissolving chitosan powder (particle size 100 nm, 99% purity, Pishgaman Co, Iran) in glacial acetic acid (Sigma-Aldrich, United States) (1% w/v) with overnight shaking at ambient temperature (25 C). The aqueous phase emulsion was formed by stirring 50 mL of chitosan solution with Tween 80 (chitosan solution:Tween 80 was 1:1.12 w/w) for 2 hr at 45 C. In 5 mL dichloromethane (Merck, Germany), different amounts of Ca ${}^{++}$ and vitamin D3 were added to form the oily phase of the emulsion: 1:0.25, 1:0.50, 1:0.75, and 1:1.00 w/w, respectively. The oily phase was homogenized with the aqueous phase at 14,000 rpm for 10 min under an ice bath on a rotor-stator homogenizer (Polytron, Kinematica, Germany). Chitosan solution polymer cross-linking was performed using the ionic gelation method. An aqueous sodium tripolyphosphate solution (0.4% w/v) was added to 50 mL of emulsion under 500 rpm stirring for 40 min at room temperature, followed by centrifugation at 10,000 rpm (4 C) for 30 min, followed by several washes with deionized water. The resultant suspension was subsequently frozen at -65 C for 72 hr. During the study, the nanoparticles were kept in the refrigerator.

The nanoparticles were orally given at a dose of 100 mg/kg immediately after the training session for 6 wk. The control supplement group received the same volume of the extract following the same procedure.

### Estrogen induction

5 μg/kg body weight of estrogen (Sigma-Aldrich, United States) was dissolved in 100 μl of corn oil and injected subcutaneously into each rat 3 times a wk for 6 wk.

### Determination of gene expression

To study the genes' expression, tissue analysis was performed using real-time polymerase chain reaction with special kits, explained in a study conducted previously by the researchers (20). First, total RNA was extracted from the liver and converted to cDNA. Then the cDNA was amplified by real-time polymerase chain reaction and the cycle threshold of the genes was recorded and converted to relative gene expression data (20). The sequence of primers is presented in table I. The procedure for measurement of dead cells was in accordance with a study conducted previously by the researchers (21).

**Table 1:** The sequence of primers used in the study


**Gene**	**Forward**	**Reverse**
***BAX***	GCAAACTGGTGCTCAAGG	CAGCCACAAAGATGGTCA
***BCL-2***	GAGTGGGATACTGGAGATGAAG	TGGTAGCGACGAGAGAAGTC
**Caspase-3**	AAGTGATGGAGATGAAGGAGT	CAGGCGTGAATGATGAAGAGT
***GAPDH***	AAGTGATGGAGATGAAGGAGT	CAGGCGTGAATGATGAAGAGT
*BAX*: BCL-2-associated X, *BCL-2*: B*-*cell lymphoma 2,* GAPDH*: Glyceraldehyde-3-phosphate dehydrogenase		

### Ethical considerations

All principles of animal care approved by the Ministry of Health and Medical Education of Iran were observed in the present study. All stages of this study were designed in accordance with the Iranian Ministry of Health for Animal Studies guidelines and were approved by the Ethical Committee of Central Tehran Branch, Islamic Azad University, Iran (Code: IR.IAU.CTB.REC.1400.063).

### Statistical analysis

All data were reported in figures and tables based on the mean and standard deviation. The healthy control group and the postmenopausal control group were compared using the independent samples *t* test. To determine the main effect of resistance training, of the drugs (vitamin D combined with calcium coated with chitosan; estrogen) and of the interaction of resistance training with the drug, two-way analysis of variance for independent groups was used. The level of significance was considered as p < 0.05. The analyses were conducted using the Statistical Package for the Social Sciences (SPSS), version 17.0 (SPSS Inc, Chicago, Illinois, USA).

## 3. Results

Following menopause induction, the gene expression of *BAX* and caspase-3, the *BAX* to *BCL-2* ratio and the percentage of dead cells of hippocampal tissue increased significantly, while the gene expression of *BCL-2* decreased significantly (Table II).

As shown in table III, resistance training significantly reduced hippocampal *BAX* gene expression. However, the expression of this gene in the training group was significantly higher than in the group with vitamin D and chitosan coating. There was no significant difference in the expression of this gene between the training group and the training + estrogen group. Drug administration also had a significant effect on hippocampal *BAX* gene expression. At the end of the period, hippocampal *BAX* gene expression was significantly higher in the estrogen group than in the vitamin D group with chitosan coating. The expression of this gene in the group of vitamin D with chitosan coating and in the group with estrogen was significantly lower than in the menopausal control group. The interaction of the 2 interventions on the expression of this gene was not statistically significant (Table III).

Resistance training significantly increased *BCL-2* gene expression in the hippocampus. Drug administration also had a significant effect on hippocampal *BCL-2* gene expression. Hippocampal *BCL-2* gene expression at the end of the period was significantly lower in the estrogen-receiving group than in the chitosan-coated vitamin D group. Expression of this gene in the vitamin D group with chitosan and in the estrogen group was significantly higher than in the menopausal control group. The training-drug interaction also had a significant effect on *BCL-2* hippocampal gene expression. In other words, the interaction between resistance training and medication (vitamin D coated with chitosan or estrogen) on *BCL-2 *gene expression was significant and had an agonistic effect. Also, the concurrence of these 2 interventions had an increasing effect on *BCL-2* gene expression (Table III).

Resistance training significantly reduced the ratio of *BAX* to *BCL-2*. Drug administration also had a significant effect on the *BAX* to *BCL-2* ratio in the hippocampus. Gene expression of *BAX* to *BCL-2* ratio in the hippocampus at the end of the period was significantly higher in the estrogen group than in the vitamin D group with chitosan coating. The ratio in the vitamin D group with chitosan and in the estrogen group was significantly lower than in the menopausal control group. The training-drug interaction had a significant effect on the *BAX* to *BCL-2* ratio in the hippocampus. The *BAX* to *BCL-2* ratio in the training + vitamin D group with chitosan coating was not significantly different from in the training + estrogen group or in the vitamin D group with chitosan coating. However, this ratio in the training + vitamin D group with chitosan coating was significantly lower than in the training, estrogen, and postmenopausal control groups (Table III).

The interaction between resistance training and medication (vitamin D coated with chitosan or estrogen) had an agonistic effect on the *BAX* to *BCL-2* ratio. The concurrence of these 2 interventions had a reducing effect on the *BAX* to *BCL-2* ratio(Table III).

Resistance training significantly reduced hippocampal caspase-3 gene expression. Drug administration also had a significant effect on hippocampal caspase-3 gene expression. Hippocampal caspase-3 gene expression at the end of the period was significantly lower in the estrogen group than in the vitamin D group with chitosan coating. The expression of this gene in the vitamin D group with chitosan and in the group with estrogen was significantly lower than in the menopausal control group. However, caspase-3 gene expression in the training + vitamin D group with chitosan coating was not significantly different from in the training + estrogen group. The expression of this gene was significantly lower in the training + vitamin D with chitosan coating group than in the training group, the vitamin D with chitosan coating group, or the estrogen group. But regarding the interaction between training and the drug, there was no significant effect on caspase-3 gene expression in the hippocampus (Table III).

Resistance training significantly reduced the percentage of dead hippocampal cells (Table III). Drug administration also had a significant effect on the percentage of dead hippocampal cells. The percentage at the end of the period in the estrogen group did not differ significantly from in the vitamin D group with chitosan coating. The percentage of dead hippocampal cells in the vitamin D group with chitosan and in the group with estrogen was significantly lower than in the menopausal control group (Table III).

The training-drug interaction had a significant effect on the percentage of dead hippocampal cells. The number of dead cells in the training + vitamin D group with chitosan coating was not significantly different from in the training + estrogen group. However, the percentage of these cells was significantly lower in the chitosan-coated vitamin D group than in the training and chitosan-coated vitamin D group and in the estrogen group (Table III). Considering this, it was concluded that the concurrence of resistance training and vitamin D with chitosan coating had a significant and agonist effect on the percentage of dead cells in hippocampal tissue. In other words, the concurrence of these 2 interventions had a reducing effect on the percentage of dead cells in the hippocampal tissue (Table III).

**Figure 1:**
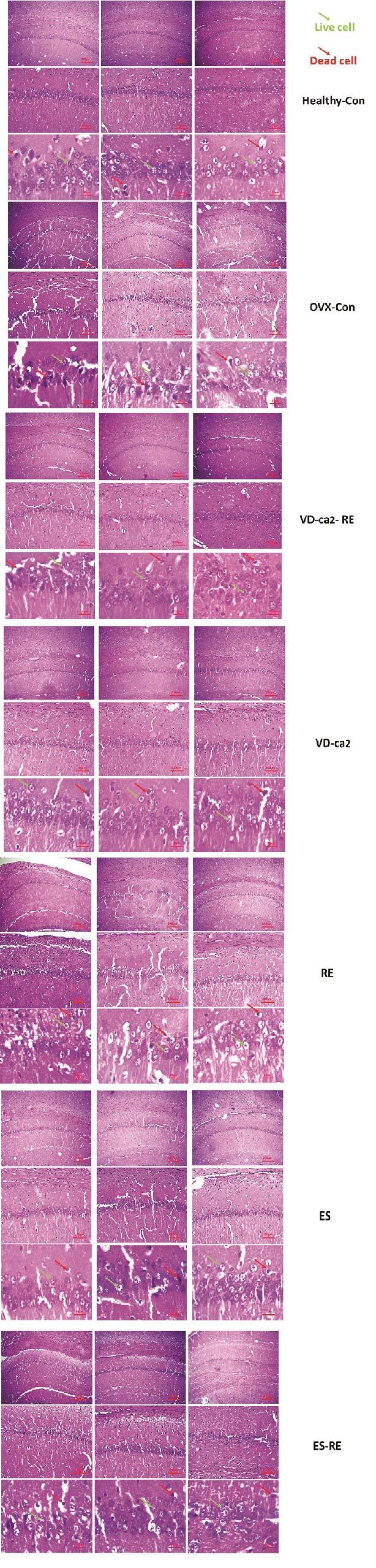
Histomorphological assessment. Coronal cross-sectional images of the hippocampus in the rat brain tissue stained with hematoxylin and eosin dyes.VD-ca2-RE: Vitamin D Ca ${}^{++}$ chitosan nanoparticles + Resistance training, ES-RE: Estrogen + Resistance training, RE: Resistance training, VD-ca2: Vitamin D Ca ${}^{++}$ chitosan nanoparticles, ES: Estrogen, OVX-Con: Ovariectomized-control.

The results of coronal cross-sectional images of the hippocampus of the rat brain tissue stained with hematoxylin and eosin dyes are shown in figure 1. Based on the images obtained from the different groups, it was found that accumulation of living neuronal cells in the CA1 region of the hippocampus was observed in the control group. In this group, only a small percentage of neurons died (up to 5%). Other parts of the hippocampus were observed to be normal. In this group, less neuronal death was observed than in the other groups. Neuronal cell death was up to 20% in the drug and training groups. Also, examining the images in the 2 groups of estrogen and vitamin D showed that accumulation of living neuronal cells in the CA1 region of the hippocampus was observed, and in these groups, only a small percentage of neurons (about 15-20%) died.

On the other hand, the results of the model group showed that different regions of the hippocampus did not have a clear coherence and order. This tissue disruption was due to removing some dead cells and apoptosis. The ratio of the cell accumulation to other groups was reduced, plus tissue cohesion was observed due to cell reduction with more disruption.

Examination of the images of the 2 groups of vitamin D + training and estrogen + training showed that the cohesion in the brain tissue somewhat improved compared to the model group. Also, the number of dead cells in the hippocampus had decreased compared to the model group, and about 10% of the cells had apoptosis.

**Table 2:** The hippocampus apoptosis biomarkers in the healthy group vs. the ovariectomized-control group


	**Healthy-control**	**Ovariectomized-control**	**P-value**
***BAX***	0.004 ± 0.0004	0.010 ± 0.0025	0.001
***BCL-2***	0.0099 ± 0.0029	0.0041 ± 0.0014	0.001
***BAX*** **:** ***BCL-2***	0.47 ± 0.20	2.60 ± 0.52	0.001
**Caspase-3**	0.0005 ± 0.0001	0.0025 ± 0.0001	0.001
**Dead cells**	6.10 ± 0.66	21.30 ± 3.89	0.001
Data are presented as Mean ± Standard deviation. There were 7 rats in each group. Data were analyzed using independent samples *t* test. *BAX*: BCL-2-associated X, *BCL-2*: B-cell lymphoma 2			

**Table 3:** Intergroup comparison of the mean of *BAX*, *BCL-2*, *BAX* to *BCL-2* ratio, caspase-3 mRNA, and dead cells


	**VD-c Ca ${}^{++}$ RT**	**ES-RT**	**RT**	**VD-c Ca ${}^{++}$ **	**ES**	**OVX-Con**
***BAX***	0.0052 ± 0.0010 ${}^{*†‡}$	0.0067 ± 0.0005 ${}^{*}$	0.0094 ± 0.0006 ${}^{*}$	0.0055 ± 0.0008 ${}^{*†‡}$	0.0087 ± 0.0009 ${}^{*}$	0.0100 ± 0.0025
***BCL-2***	0.0085 ± 0.0010 ${}^{*†‡}$	0.0088 ± 0.0010 ${}^{*}$	0.0065 ± 0.0010 ${}^{*}$	0.0070 ± 0.0009 ${}^{*†‡}$	0.0052 ± 0.0010 ${}^{*}$	0.0041 ** ± ** 0.0010
***BAX*** **: ** ***BCL-2***	0.62 ± 0.28 ${}^{*†‡}$	0.79 ± 0.15 ${}^{*}$	1.50 ± 0.42 ${}^{*}$	0.69 ± 0.30 ${}^{*†‡}$	1.70 ± 0.32 ${}^{*}$	2.60 ± 0.53
**Caspase-3**	0.0009 ± 0.0002 ${}^{*†‡}$	0.0013 ± 0.0003 ${}^{*}$	0.0016 ± 0.0002 ${}^{*}$	0.0014 ± 0.0003 ${}^{*†‡}$	0.0019 ± 0.0002 ${}^{*}$	0.0020 ± 0.0001
**Dead cells**	9.80 ± 1.02 ${}^{*†‡}$	7.90 ± 0.66 ${}^{*}$	20.20 ± 1.02 ${}^{*}$	16.00 ± 1.41 ${}^{*†‡}$	16.60 ± 1.46 ${}^{*}$	21.30 ± 3.89
Data are presented as Mean ± Standard deviation. There were 7 rats in each group. Data were analyzed using a two-way analysis of variance with post hoc test. P < 0.05 was considered as a significant effect. *BAX*: BCL-2-associated X, *BCL-2*: B-cell lymphoma 2, VD-c Ca ${}^{++}$ RT: Vitamin D Ca ${}^{++}$ chitosan nanoparticles + Resistance training, ES-RT: Estrogen + Resistance training, RT: Resistance training, VD-c Ca ${}^{++}$ : Vitamin D Ca ${}^{++}$ chitosan nanoparticles, ES: Estrogen, OVX-Con: Ovariectomized-control. ${}^{*}$ Significant difference with the OVX-Con group. ${}^{‡}$ Significant difference with the RT group. ${}^{†}$ Significant difference with the ES group						

## 4. Discussion

The results of the present study showed that OVX induced an increase in the gene expression of *BAX* and caspase-3, the *BAX* to *BCL-2* ratio and the percentage of dead cells of hippocampal tissue, while the gene expression of *BCL-2* decreased. Estrogen deficiency leads to the release of free radicals and reactive oxygen species, and causes oxidative stress and some metabolic diseases. Oxidative stress can lead to mutations in the mitochondrial genome and subsequent cell death through necrosis or apoptosis. Several studies have investigated the effect of OVX on apoptosis-related factors. In cardiac tissue, OVX increases cholesterol, triglycerides, low-density lipoprotein, *Bax*, caspase-3, and caspase-8, and decreases high-density lipoprotein, miR-133, and *BCL-2* (22). Caspase-3 is key for apoptosis because it regulates part of the cell apoptosis pathway. *BCL-2* is essential for apoptosis since it stops cell passing, and the gene expression of *BAX* is recognized as the primary part of the *BCL-2* family in line with apoptosis (23).

Exercise prevents mitochondrial-dependent apoptosis, which is characterized by expanded expression of *BCL-2*, diminished expression of *BAX*, a diminished *BAX* to *BCL-2* ratio, diminished caspase-9 action, and inactivated caspase-3 (24). Exercise prevents apoptosis via the mitochondrial-related pathway. The *BCL-2* family proteins intercede the mitochondrial pathway of apoptosis, which anticipates the release of cytochrome C from the mitochondria (24). Enhanced *BAX/BCL-2* antagonist/killer, reactive oxygen species and calcium particles lead to the release of cytochrome C from the mitochondria into the cytosol. Consequently, exercise appears to be useful in avoiding cardiac apoptosis by lessening reactive oxygen species and avoiding the release of mitochondrial cytochrome C (25). The results of the present study showed that resistance training reversed the negative effects of OVX on *BAX*, *BCL-2*, the *BAX* to *BCL-2* ratio, and caspase-3 expression in the hippocampus tissue of rats.

Huang and colleagues investigated the antiapoptotic effect of exercise training on the heart of OVX rats. The results of their study in OVX rats showed that the protein levels of several factors including tumor necrosis factor-α, tumor necrosis factor receptor 1, Fas ligand, Fas receptors, Fas*-*associated death domain, activated caspase-8 and activated caspase-3, truncated *p15 BID*, *BCL-2-*associated agonist of cell death, *Bak*, *Bax*, cytosolic cytochrome c, activated caspase-9, and activated caspase-3 decreased responses to exercise (26). Habibi and colleagues showed that exercise training attenuated diabetes-induced cardiac injury through increasing miR-133a and improving pro-apoptosis/anti-apoptosis balance in OVX rats. They showed that exercise training significantly reversed the OVX-induced change in *Bax*, caspase-3, caspase-8, high-density lipoprotein, cholesterol, triglyceride, and miR-133 (22).

In addition, the results of the present study showed that taking vitamin D Ca ${}^{++}$ chitosan nanoparticles modulates the negative effects of OVX on *BAX*, *BCL-2*, the *BAX* to *BCL-2* ratio, and caspase-3 expression in the hippocampus tissue of rats. Few studies have examined the effect of vitamin D or chitosan intake on *BAX*, *BCL-2*, or caspase-3 in menopause conditions. The paracrine feedback mechanism of vitamin D can reduce the inflammatory response. This is how it affects our immune system. Taking supplements such as vitamin D and calcium positively affects apoptosis in menopausal conditions. Vitamin D can modulate apoptosis-related components in menopausal conditions by inhibiting apoptotic proteins or stimulating anti-apoptotic proteins. It has been reported that vitamin D supplements may have anti-inflammatory effects against eccentric exercise-induced delayed onset muscle soreness in female students (27). Zou and colleagues showed that adriamycin induced apoptosis through an imbalance of phospho-Smad2/3, p-Smad1/5/8, and the activity of caspase-3, and aberrant expression of Fas, Fas-associated via death domain, *BAX*, and *BCL-2*. Vitamin D adjusts these harmful imbalances (28). Acetylated chitosan oligosaccharides have been reported to act as antagonists against glutamate-induced PC12 cell death via the *BCL-2/BAX* signaling pathway (19). It has also been shown that curcumin-containing chitosan nanoparticles have antioxidant and proapoptotic effects via impact on caspase-3 gene expression (29). In addition, it has been shown that chitosan induces apoptosis activation in tumor cells (30).

One of the limitations of the present study was the lack of investigation of different doses of this supplement. Therefore, further research is needed to examine different doses of the supplements. Also, since measuring gene expression is not a definitive indication of protein conversion, performing future studies with methods such as Western blotting could be useful.

## 5. Conclusion

Based on the results of the present study, the combined effect of resistance training and vitamin D Ca ${}^{++}$ chitosan nanoparticle supplementation had a similar effect as estrogen on apoptosis improvement in the conditions of menopause. Thus, a combination of resistance training and vitamin D Ca ${}^{++}$ chitosan nanoparticle supplements may be considered a non-pharmacological treatment to treat OVX-induced apoptosis. Using higher doses of this natural supplement may have the same or even better positive effects than estrogen.

##  Conflict of Interest

The authors declare that there is no conflict of interest.
